# Regulation of Bone Marrow Angiogenesis by Osteoblasts during Bone Development and Homeostasis

**DOI:** 10.3389/fendo.2013.00085

**Published:** 2013-07-10

**Authors:** Ernestina Schipani, Collen Wu, Erinn B. Rankin, Amato J. Giaccia

**Affiliations:** ^1^Division of Endocrinology, Department of Medicine, Indiana University Medical School, Indianapolis, IN, USA; ^2^Division of Radiation and Cancer Biology, Department of Radiation Oncology, Stanford University School of Medicine, Stanford, CA, USA

**Keywords:** hypoxia-inducible factor, vascular endothelial growth factor, prolyl hydroxylases, erythropoietin, osteoblasts

## Abstract

Bone marrow is a highly heterogeneous and vascularized tissue. The various cell types populating the bone marrow extensively communicate with each other, and cell-to-cell cross talk is likely to be essential for proper bone development and homeostasis. In particular, the existence of osteogenesis and angiogenesis coupling has been recently proposed. Despite its high degree of vascularization, a gradient of oxygenation is present in the bone marrow, and the endosteal surface of cortical bone appears to be among the most hypoxic areas in the body. Oxygen (O_2_) is both an essential metabolic substrate and a regulatory signal that is in charge of a specific genetic program. An important component of this program is the family of transcription factors known as hypoxia-inducible factors (HIFs). In this Perspective, we will summarize our current knowledge about the role of the HIF signaling pathway in controlling bone development and homeostasis, and especially in regulating the crosstalk between osteoblasts, progenitor cells, and bone marrow blood vessels.

## Bone Marrow: A Highly Vascularized Tissue with a Gradient of Oxygenation

Skeletal development is dependent on two mechanisms: intramembranous ossification and endochondral replacement (1, 2). Intramembranous ossification is a process in which mesenchymal cells differentiate directly into osteoblasts, is responsible for the formation of the flat bones of the skull. In contrast, endochondral replacement, which accounts for the development of most other bones, is a two-step process: mesenchymal cells give origin to chondrocytes, which build a template known as growth plate; this template is next replaced by bone.

Bone marrow is a highly heterogeneous and highly vascularized tissue. In addition to endothelial cells, at least two other cell types populate the adult bone marrow: hematopoietic cells, which originate from the very well characterized hematopoietic stem cell (HSC), and mesenchymal cells, which include stromal cells, osteoblasts, and adipocytes, and are thought to be derived from a still not fully defined mesenchymal stem cell population (3). Stromal cells are a meshwork of osteoblast precursors and specialized fibroblasts known as reticular or adventitial cells. However, the relationship between these two types of stromal cells, if any, remains to be elucidated (4).

It is noteworthy that HSCs can also give rise to osteoclasts, which are specialized cells that resorb bone (5). Furthermore, it has been hypothesized that hematopoietic and endothelial cells probably share a common mesodermal precursor (6).

The clear dichotomy between hematopoietic-endothelial cells and mesenchymal cells and their distinct embryological origin was already recognized more than 40 years ago by Le Douarin, who wrote in one of her seminal papers “*The contribution to bone marrow histogenesis of cells of vascular and blood origin, on one hand, and of the elements of the cartilaginous model, on the other hand, was analyzed. It appeared that osteoblasts, osteocytes, and stromal cells of marrow are derived from the perichondrium. In contrast, the endothelium of the vascular buds and the hemopoietic cells which invade the diaphyseal cartilage during the endochondral ossification process do not belong to the mesenchymal bone primordium but have a fully extrinsic origin*” (7).

The various cell types populating the bone marrow extensively communicate with each other, and the cellular cross talk is essential for proper control of bone and bone marrow development and homeostasis. For example, endothelial cells, osteoblasts, and stromal cells are components of niches that have an important role in the maintenance of the HSC pool (8–13), and in the regulation of the number of B-lymphocytes (14–16) and granulocytes (15, 17). Conversely, hematopoietic cells, in particular mast cells and megakaryocytes, are known to produce a large set of cytokines and growth factors that significantly affect bone mass (18, 19).

The capillary bed in the bone marrow is formed by “sinusoidal” capillaries (20–22). Sinusoids are discontinuous capillaries as their endothelial cells have both large fenestrations and wide spaces among them (23). Moreover, their basal membrane is also discontinuous. Their diameter is large (30–40 μm), which considerably reduces the rate of blood flow (24). These unique anatomical and functional features allow for maximum exchange of macromolecules as well as cell movement between the bone marrow and the blood. Sinusoidal capillaries are also found in liver and spleen (23), namely in the other two major organs that at different physiological stages and in pathological conditions contribute to hematopoiesis. Mesenchymal cells known as pericytes intermittently surround the sinusoidal endothelial cells (4), and produce their own basal membrane, which blends with the basal membrane produced by the endothelium.

O_2_, nutrients, and a cornucopia of hormones and growth factors, which are important for bone and bone marrow development and homeostasis, are brought to bone by blood vessels (25).

In addition, it has been convincingly proven that blood vessels supply osteoblast precursors either with their flow (26) or as components of their walls (4, 27). Along these lines, it has been shown that bone marrow pericytes, which are positive for CD146 contribute to colony forming units *in vitro* and thus, according to the model proposed by Friedenstein decades ago (28, 29), are likely precursors of osteoblasts and bone marrow adipocytes (4). Moreover, it has been elegantly demonstrated that blood vessels guide the migration of osteoblast precursors from the periosteum to the bone marrow (27). Conversely, fully differentiated periosteal osteoblasts have apparently lost the ability to migrate from the periosteum into the bone marrow (27). Lastly, it has been reported that endothelial cells expressing mutant activin 1 receptors (ACVR1) can trans differentiate into osteoblasts, an event that may be critical in the pathogenesis of a devastating disease such as Fibrodysplasia Ossificans Progressiva (FOP) (30–32).

Taken together, the concept that blood vessels regulate the pool of osteoblasts both in physiological and in pathological states is progressively gaining strong support by the scientific community (33). Whether and how cells of the osteoblast lineage control number and shape of blood vessels in physiological conditions *in vivo*, is also an important question that needs to be investigated in more depth.

Despite its high degree of vascularization, a gradient of oxygenation is present in the bone marrow, and the endosteal surface of cortical bone is among the most hypoxic areas as revealed by staining with the marker of hypoxia pimonidazole (34–36). The high degree of bone marrow cellularity, the high levels of O_2_ consumption by leukocytes as well as the sluggish blood flow in the sinusoids are all thought to be all contributing factors to the generation of a gradient of oxygenation within the bone marrow (36, 37).

In the next sections of this Perspective, we will discuss the most recent findings about the role of the hypoxia-inducible factor (HIF) signaling pathway in osteoblast biology and in the osteoblast-dependent control of bone marrow angiogenesis.

## The HIF Signaling Pathway

Oxygen is both an essential metabolic substrate in numerous enzymatic reactions, including mitochondrial respiration, and a regulatory signal that controls a specific genetic program. An important component of this program is the transcription factor HIF-1α (2, 38–43), which is a key-mediator of cellular adaptation to low O_2_ tension (hypoxia). In normoxia, a class of 2-oxoglutarate-dependent and Fe^2+^-dependent prolyl-4-hydroxylases (PHDs) hydroxylate specific proline residues of the HIF-1α protein (44, 45). Hydroxylated HIF-1α is then targeted to the proteosome for degradation by the E3 ubiquitin ligase von Hippel Lindau (VHL). Under hypoxic conditions, hydroxylation of HIF-1α diminishes, HIF-1α protein accumulates, translocates from the cytoplasm to the nucleus, dimerizes with its constitutively expressed partner HIF-1β, binds in a sequence dependent manner to hypoxia-responsive elements, recruits transcriptional co-activators, and increases the transcription of an ever growing number of hypoxia-responsive genes (44–49). The protein products of these HIF target genes regulate a variety of biological processes, including angiogenesis, non-oxidative glycolysis, and matrix formation (2). While the mRNA encoding HIF-1α is widely expressed (50), the regulation of HIF-1α is largely post-transcriptional. In tissues where oxygen tension is higher than 5%, HIF-1α protein is barely detectable, but when oxygen tension drops below 5% HIF-1α protein progressively accumulates. Mouse embryos lacking HIF-1α die *in utero* by E11 (51–53), indicating that HIF-1α is critical for embryonic development.

Two other members of the family, HIF-2α and HIF-3α, have been cloned and characterized. Hypoxia controls stability of HIF-2α in a similar fashion as HIF-1α (54). HIF-2α and HIF-1α have common targets as well as specific ones (54–58). Moreover, they exhibit different tissue expression patterns (59). Lastly, in some genetic backgrounds mice lacking HIF-2α survive postnatally (60), where lack of HIF-1α causes early embryonic lethality. In contrast to HIF-1α and HIF-2α, the biological role of the HIF-3α isoform is still largely unknown, though it has been proposed that this protein could have a dominant negative function, since it lacks transactivation domains (61).

The E3 ubiquitin ligase VHL is expressed in most tissues and cell types, and is critical for HIF-1α degradation. Heterozygous missense mutations of the *VHL* gene have been identified in the VHL syndrome (62). This syndrome is characterized by a dominantly inherited predisposition to develop pheochromocytoma, clear cell renal carcinoma, and hemangioblastomas in the central nervous system and in the retina (62). As discussed above, VHL has E3 ubiquitin ligase activity, and the HIFs are among its main targets. The importance of VHL for proteolysis of HIF-1α and HIF-2α is highlighted by the finding that cells lacking a functional VHL are unable to degrade these transcription factors, ultimately resulting in an accumulation of HIFs (45, 49, 63, 64). However, VHL has also a variety of biological activities including control of the cell cycle, critical interaction with the cytoskeleton and with the primary cilia, and regulation of matrix proteins that have been reported to be HIF-independent (62).

## The HIF Signaling Pathway in Osteoblasts and Its Impact on Bone and Blood Vessels

Loss of VHL in fully differentiated osteoblasts, resulting in the stabilization of HIF-1α and HIF-2α, and increased activity of HIF signaling in these cells, leads to a high bone mass phenotype in the bones that are formed through the endochondral replacement process. In contrast, the flat bones of the skull, which develop through an intramembranous program, appear to be largely unaffected (36, 65). Strikingly, this increased accumulation of trabecular bone in the long bones is associated with an augmented surface and volume of blood vessels in the bone marrow cavity (16, 65). Therefore, activation of the HIF signaling pathway in osteoblasts is sufficient to modulate bone marrow angiogenesis.

**Figure 1:**
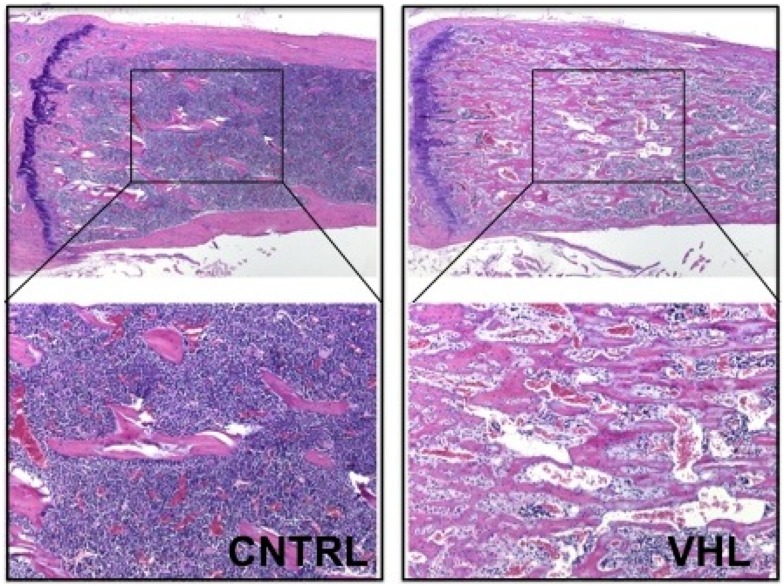
**Loss of VHL in cells of the osteoblast lineage increases trabecular bone mass**. H and E of histological sections of tibia isolated from 4-month-old control (CNTRL) and OSX-VHL mutant (VHL) mice; bottom panels are blows up of a and b, respectively. For details, see Text.

More recently, these findings were confirmed and expanded in another mouse model lacking VHL in osteoprogenitors. Loss of VHL in osteoprogenitors (OSX-VHL) caused a dramatic increase of trabecular bone mass (16). In particular, OSX-VHL mutant mice exhibited excessive accumulation of trabecular bone in the metaphyseal and diaphyseal regions of the long bones (16). Surrounding the numerous trabecule were abundant stromal cells and strikingly dilated blood vessels (angiectasis) (Figure 1) (16). In agreement with previous findings, the frequency of HSCs was increased in the bone marrow of OSX-VHL mutant mice (16). Although it is still uncertain whether this apparent expansion of the HSC pool is a consequence of either the expanded osteoblastic niche, or the augmented blood vessel surface, or both. Dilated blood vessels and expansion of stromal cells are two histological hallmarks of the hemangioblastoma lesions identified in VHL syndrome (66). Loss of HIF-1α and HIF-2α in VHL-deficient osteoprogenitors fully corrects the high bone mass phenotype, the increased number of HSCs, the angiectasis, and the aberrant expansion of the bone marrow stroma secondary (16).

Taken together, these data demonstrate that augmented HIF activity in osteoprogenitors augments trabecular bone volume, increases the bone marrow stromal population, and expands the HSC pool (16). Moreover, loss of VHL in osteoprogenitor cells demonstrates that stabilization of HIFs in osteoblasts is sufficient to increase number and/or size of blood vessels in the bone marrow. However, it remains unresolved whether osteoblastic HIFs are necessary for regulation of bone marrow angiogenesis in a more physiological setting.

## VEGF and EPO: Potential Candidates Mediating the Osteoblasts-Blood Vessels Cross Talk in the Bone Marrow

Numerous proangiogenic factors are transcriptionally regulated by HIFs, and each of them could be responsible for the modulation of blood vessel number and/or size by osteoblasts when the HIF transcriptional pathway is activated in these cells. Among them, vascular endothelial growth factor-A (VEGF) is one of the best-characterized downstream targets of HIFs.

Vascular endothelial growth factor is a glycoprotein that belongs to the dimeric cysteine-knot growth factor super-family (67, 68). The mouse VEGF gene encodes at least three isoforms (VEGF120, VEGF164, and VEGF188) that arise through alternative splicing (69, 70). While VEGF164 and VEGF188 bind the extracellular matrix component heparan sulfate, VEGF120 lacks the binding motif necessary for this interaction (71, 72). VEGF is a principal regulator of blood vessel formation, and it is also a survival factor for HSCs *in vivo* and *in vitro*, through an internal autocrine loop mechanism (73). In endochondral bones, VEGF is a survival factor for chondrocytes *in vivo*, at least in part by regulating angiogenesis in the surrounding soft tissue (74). Moreover, in hypertrophic chondrocytes it controls blood vessel invasion and thus replacement of cartilage by bone *in vivo* (75). Furthermore, it promotes osteogenesis *in vitro* (68), and when overexpressed in cells of the osteoblast lineage *in vivo* it leads to a high bone mass phenotype associated to marrow fibrosis and increased number of blood vessels (76). These effects appear to be secondary, at least in part, to activation of beta catenin in osteoblasts (76). Conversely, mice that lack VEGF in osteoprogenitors display a reduced bone mass phenotype with increased bone marrow fat. Even more intriguing in this mouse model, VEGF regulates the balance between osteoblasts and adipocytes by a novel, intracrine mechanism (77). VEGF is also a positive modulator of osteoclastogenesis as well, both *in vivo* and *in vitro* (76, 78, 79).

Vascular endothelial growth factor expression, as expected, is increased in mutant bones lacking VHL in cells of the osteoblast lineage (16, 65). Therefore, it is reasonable to hypothesize that VEGF could be responsible for the increased number and/or size of blood vessels in these mutants (25), although this possibility has still to be experimentally tested.

Notably, mutant mice lacking VHL in cells of the osteoblast lineage develop severe polycythemia, which is due to HIF-2α dependent overproduction of erythropoietin (EPO) by osteoblasts (16). This study provided the first demonstration that cells of the osteoblast lineage are capable of producing and secreting EPO upon activation of the HIF signaling pathway *in vivo* (16). The HIF-2, rather than the HIF-1, dependence of EPO production is consistent with what has been reported in other experimental models (80, 81). EPO is a hormone that stimulates erythropoiesis through binding and activation of its receptor (EpoR) in erythroid progenitor cells (82–84). In adults, more than 90% of EPO is produced in the kidney by a subset of peritubular interstitial fibroblasts (84). The key regulatory mechanism of EPO expression is hypoxia through the activation of the HIF pathway (84). Anemia stimulates EPO production by inducing local hypoxia (84). Unlike EPO, EpoR expression is not affected by hypoxia or anemia (83). In the cell membrane, EpoR forms a homodimer that, upon EPO binding, recruits JAK2, which in turns leads to activation of STAT5, PI3 Kinase (PI3K), and Ras-MAPK signaling pathways (83). Both EPO and EpoR are also expressed in a variety of non-hematopoietic tissues, which implies that EPO may have functions that go beyond regulation of erythropoiesis. For example, EpoR has been identified on the surface of endothelial cells (82, 83). Consistent with this finding, the EPO/EpoR complex is thought to be an important regulator of angiogenesis, particularly in the context of malignant tumors (82, 83). Interestingly, in addition to its direct effect on endothelial cells, EPO may control angiogenesis by modulating the release of other angiogenic factors such as VEGF (83).

**Figure 2:**
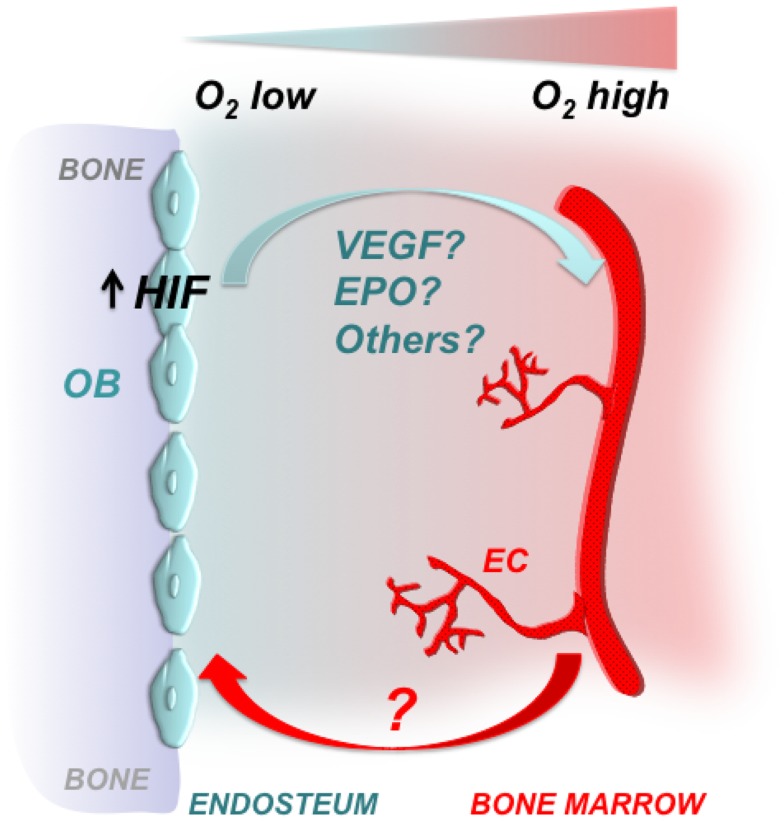
**Osteoblastic HIFs control bone marrow angiogenesis**. Gradients of oxygenation are present in the bone marrow with osteoblasts at the endosteal surface of bone being among the most hypoxic areas. Osteoblasts express HIFs, which regulate osteoblast activity and angiogenesis. Some of the HIF effects on angiogenesis could be mediated by VEGF, EPO, and/or other proangiogenic factors. It has yet to be defined whether increased angiogenesis is a necessary pre-requisite for the high bone mass phenotype observed upon activation of HIFs in cells of the osteoblast lineage. OB, osteoblast; EC, endothelial cell.

Taken together, it is tempting to speculate that both EPO and VEGF interact to coordinate bone marrow angiogenesis, when the HIF signaling pathway is activated in osteoblasts (Figure 2).

## Future Directions

In this Perspective, we have highlighted the critical role of the HIF signaling pathway in regulating bone mass and bone marrow angiogenesis. It will be now important to identify the molecular mechanisms that mediate osteoblasts-blood vessel cross talk, and to define whether increased angiogenesis is a necessary pre-requisite for the high bone mass phenotype observed upon activation of HIFs in cells of the osteoblast lineage (Figure 2). Addressing these two questions may significantly expand our understanding of the osteogenesis–angiogenesis coupling in physiological and pathological states.

## Conflict of Interest Statement

The authors declare that the research was conducted in the absence of any commercial or financial relationships that could be construed as a potential conflict of interest.
